# An Enzymatic Biosensor for the Detection of D-2-Hydroxyglutaric Acid in Serum and Urine

**DOI:** 10.3390/bios12020066

**Published:** 2022-01-25

**Authors:** Bo Wu, Zehua Li, Zepeng Kang, Chunling Ma, Haiyan Song, Fuping Lu, Zhiguang Zhu

**Affiliations:** 1 Key Laboratory of Industrial Fermentation Microbiology, Ministry of Education, Tianjin University of Science and Technology, No.9, 13th Avenue, Tianjin Economic and Technological Development Area, Tianjin 300457, China; wub@tib.cas.cn (B.W.); lfp@tust.edu.cn (F.L.); 2 Tianjin Key Laboratory of Industrial Microbiology, The College of Biotechnology, Tianjin University of Science and Technology, No.9, 13th Avenue, Tianjin Economic and Technological Development Area, Tianjin 300457, China; 3 Tianjin Institute of Industrial Biotechnology, Chinese Academy of Sciences, 32 West 7th Avenue, Tianjin Airport Economic Area, Tianjin 300308, China; lizeh@tib.cas.cn (Z.L.); kangzp@tib.cas.cn (Z.K.); machl@tib.cas.cn (C.M.); songhy@tib.cas.cn (H.S.); 4 University of Chinese Academy of Sciences, 19A Yuquan Road, Shijingshan District, Beijing 100049, China; 5 National Technology Innovation Center of Synthetic Biology, Tianjin 300308, China

**Keywords:** D-2-hydroxyglutaric acid, MXene, biosensor, screen-printed electrode, D-2-hydroxyglutarate dehydrogenase

## Abstract

D-2-hydroxyglutaric acid (D2HG) is overproduced as a result of the D-2-hydroxyglutaric aciduria and relevant cancers, caused by gene mutation. Accurate analysis of D2HG could help rapid diagnosis of these diseases and allow for timely treatment. In this work, a D-2-hydroxyglutarate dehydrogenase from *Ralstonia solanacearum* (*Rs*D2HGDH) is cloned and recombinantly expressed. This enzyme features the direct electron transfer to chemical electron mediators (such as methylene blue (MB)) in the absence of additional coenzymes. Therefore, NAD^+^, a natural electron acceptor for the commercial D2HGDH and usually known for being unstable and difficult for immobilization can be avoided in the preparation of biosensors. The *Rs*D2HGDH and MB are co-immobilized on a two-dimensional material, Ti_3_C_2_ MXene, followed by drop-coating on the gold screen-printed electrode (AuSPE) to construct a compact and portable biosensor. The D2HG in samples can be catalyzed by *Rs*D2HGDH, where the current change is measured by chronoamperometry at −0.23 V. The biosensor shows a D2HG detection range of 0.5 to 120 µM (R^2^ = 0.9974) with a sensitivity of 22.26 μA mM^−1^ cm^−2^ and a detection limit of 0.1 µM (S/N = 3). The biosensor retains 72.52% performance of its incipient state after 30 days of storage. The samples of D2HG-containing fetal bovine serum and artificial urine were analyzed with the recovery of 99.56% to 106.83% and 97.30% to 102.47% further indicating the great application potential of our portable D2HG biosensor.

## 1. Introduction

2-hydroxyglutaric acid (2HG), also known as α-hydroxyglutaric acid (αHG), could be divided into D/L2HG according to optical rotation [1]. D2HG is one of the trace metabolic byproducts in human cells, mainly produced in the transformation process of 4-hydroxybutyrate to succinic acid semialdehyde by hydroxyacid-oxoacid-transhydrogenase [2,3]. Generally, D2HG can be oxidized to α-ketoglutarate (α-KG) under the catalysis of D2HG dehydrogenase (D2HGDH) [4]. However, the gene mutation of enzymes related to D2HG metabolism (glutaryl coenzyme A dehydrogenase, isocitrate dehydrogenase (IDH), and D2HGDH) would result in 100-fold increase of D2HG in biological fluids and cause the D-2-hydroxyglutaric aciduria (D2HGA) [5]. Additionally, mutations R132 and R172 in IDH complex are involved in multiple cancers, such as glioma [6], intrahepatic cholangiocarcinoma [7], chondrosarcoma [8], and acute myelocytic leukemia [9,10]. Briefly, the D2HG level, as a specific indicator of IDH-mutated diseases [11], is of great importance to the diagnosis of D2HGA and relevant cancers.

Many analytical methods of D2HG have been developed, including liquid/gas chromatography-mass spectrometry (LC/GC-MS) and magnetic resonance spectroscopy (MRS) [11,12,13,14]. Nevertheless, these methods always suffer from the dependence on sophisticated instruments, cumbersome operation, complex pretreatment of samples, and expensive testing cost. Recently, the colorimetry and fluorometry methods for the analysis of D2HG have been exploited [15,16]. The former employs NAD+-dependent D2HGDH to catalyze the dehydrogenation of D2HG to produce colored intermediates. However, commercial kit (for example, Abcam ab211070) based on this is limited by insufficient detection limit [17,18]. The latter method coupling resazurin analogs to quantitate D2HG through the generation of fluorophores allows for a great improvement in accuracy and detection limit. However, it suffers from the complex procedure, stringent assay environment, and long assay time. Despite this, as far as we know, the research on detection of D2HG in vitro by the biosensor for disease diagnosis is insufficient, especially the electrochemical enzymatic biosensor has never been developed.

Electrochemical enzymatic biosensor, as a fast and convenient analytical tool, catalyzes target molecules through oxidoreductase and converts chemical concentration into electrical signal [19,20,21,22,23]. With advantages of simple operation, low manufacturing cost, and minimal requirement for sample pretreatment, it has been extensively used in the field of industry, environment, and medicine [24,25,26,27]. In particular, in daily blood glucose monitoring, the biosensor based on glucose oxidase is regarded as the main analysis tool [28,29,30,31]. In general, electrochemical enzymatic biosensors are usually composed of enzymes as the key sensing element and electrodes for electron transfer. In addition, in order to boost biosensor performance and enhance the conductivity of electrodes, various nanomaterials are introduced as immobilized carriers of the enzyme. Among them, carbon nanomaterials (e.g., carbon nanotubes and graphene) were widely used benefit from their good conductivity and large specific surface area [32,33]. However, it is difficult for many carbon nanomaterials to disperse well in the water because of their hydrophobic property, which hindered the immobilization of the enzyme on the nanomaterials. Recently, the novel two-dimensional transition metal carbides and nitrides (MXene) have emerged in biosensor construction, featuring its excellent metal conductivity, stable chemical properties, and especially high hydrophilicity [34,35,36]. According to previous studies, the biosensors based on MXene have been used for the assay of phenol, cholesterol, β-hydroxybutyric acid, and prostate-specific antigen [37,38,39,40]. Therefore, it might be feasible to use MXene as a reliable decorating nanomaterial for the fabrication of a D2HG biosensor.

**Scheme 1:**
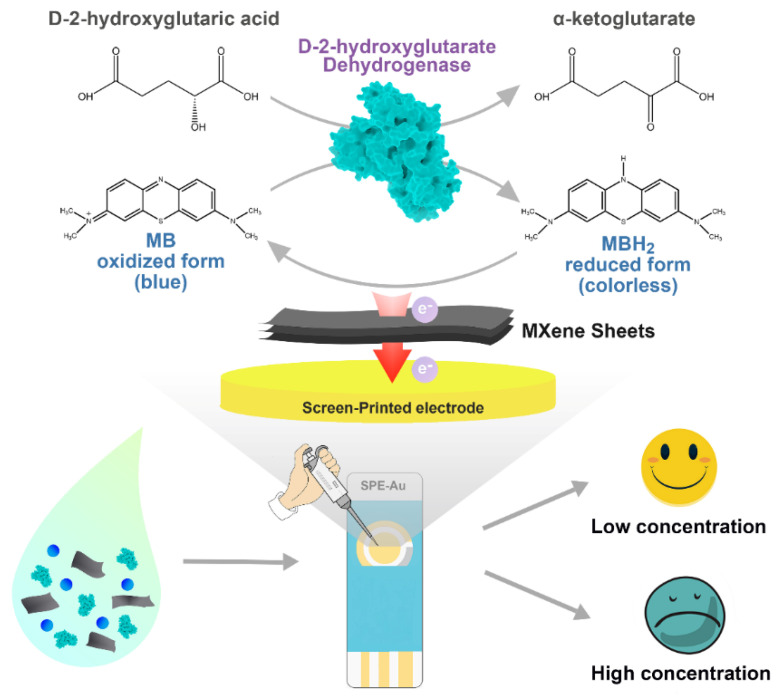
The fabrication and the working mechanism of the D2HG biosensor.

Herein, we construct a portable and sensitive electrochemical enzymatic biosensor based on MXene for the analysis of D2HG (Scheme 1). *Rs*D2HGDH, as the sensing element, and methylene blue (MB), as the electron mediator, are co-immobilized on the MXene sheets, which are further coated onto an AuSPE to fabricate the biosensor. In the presence of D2HG, *Rs*D2HGDH catalyzes the dehydrogenation of D2HG to α-KG and the generated electrons are transferred to the electrode via MB. The oxidation current of MBH_2_ is captured at the appropriate potential and linearly correlates with the concentration of D2HG in the range of 0.5 μM to 120 μM, which include the typical value of D2HG in human serum and urine [41]. Meanwhile, the performance of biosensors is systematically evaluated. Furthermore, the detection of D2HG in fetal bovine serum and in artificial urine samples is successfully conducted, suggesting satisfactory accuracy and reproducibility of the biosensor.

## 2. Materials and Methods

### 2.1. Apparatus and Reagents

Lithium fluoride (LiF), MB, potassium ferricyanide, and dopamine (DA) were purchased from Macklin (Shanghai, China). Urea, uric acid (UA), ascorbic acid (AA), and chitosan (Chi) were purchased from Aladdin (Shanghai, China). Ti_3_AlC_2_ powder (600 mesh) was purchased from Beike 2D materials Co., Ltd (Beijing, China). Phenazine methosulphate (PMS) and 2,6-dichloroindophenol (DCIP) were purchased from Sigma-Aldrich (St. Louis, MO, USA). D2HG was purchased from MedChemExpress (Monmouth Junction, NJ, USA). Hydrochloric acid, potassium ferrocyanide, glucose, and L-lactic acid were purchased from a local reagent company. Gold screen-printed electrodes (AuSPE) were purchased from Huiquan Electronics Co., Ltd (Jinan, China). The serum samples were generously donated by Beijing Municipal Health Commission (Beijing, China). The artificial urine was purchased from Beijing Leagene biotech. Co., Ltd (Beijing, China).

The images of scanning electron microscopy (SEM) were observed with JSM-7610f field emission SEM instrument (JEOL, Japan). Hitachi HT7700 was used to perform transmission electron microscopy (TEM). Bruker D8 ADVANCE A25X was used to measure the X-ray diffraction (XRD) in the scan range of 2θ = 5−80° with a speed of 5° min^−1^. Thermo ESCALAB 250XI was used to obtain X-ray photoelectron spectroscopy (XPS) spectrum. The absorbance value was determined with a Cary 100 UV-Vis spectrophotometer (Agilent Technologies Inc., Palo Alto, California, USA). Freeze drying was carried out with an FD-1A-50 freeze dryer (Shanghai Grows Instrument, Shanghai, China). The CHI 660E electrochemical workstation was used to execute the electrochemical measurements (Shanghai Chenhua Instrument, Shanghai, China).

### 2.2. Synthesis of MXene

In this study, the hydrofluoric acid (HF) was used as an etchant of the Ti_3_AlC_2_ to remove the Al layer for ultrasonic stripping of the Ti_3_C_2_ sheets [42]. Firstly, 0.5 g LiF was mixed with 10 mL 75% (*V*/*V*) HCl, before the 0.5 g Ti_3_AlC_2_ was added. Then the mixture was stirred in 35 °C for 24 h. The mixture after etching was washed with Milli-Q water until the pH was close to 6. After that, MXene sheets were dispersed by ultrasonic treatment with a nitrogen atmosphere. Finally, the larger particles were removed by centrifugation at 3500 rpm for 1 h before the supernatant was freeze-dried and stored at −20 °C.

### 2.3. Preparation and Assays of RsD2HGDH

The gene of *Rs*D2HGDH was synthesized and the codon was optimized by GenScript (Nanjing, China). The pET20b-based recombinant plasmid containing the *Rs*D2HGDH gene was transformed into an expression strain (*E. coli* BL21) and 0.01 mM isopropyl-β-D-thiogalactopyranoside was used to induce enzyme expression at 16 °C when the OD_600_ was about 0.6. The cells were washed with 20 mM phosphate buffer (PB, pH 7.0) and broken by ultrasound. The broken cells were centrifuged at 8000 rpm for 15 min to obtain crude enzyme solution. The supernatant was purified through the His-tag adsorbed on the Ni-NTA resin and eluted by 20 mM PB containing 200 mM imidazole. Finally, ultrafiltration was used to concentrate purified enzymes and remove imidazole.

The activity of *Rs*D2HGDH was determined by detecting the absorbance of DCIP at 600 nm [43]. The assay mixture (500 μL) comprised 100 µM DCIP, 200 µM PMS, 50 mM PB (pH 7.0) and 5 mM D2HG. One unit of enzyme activity was defined as the amount of enzyme catalyzing the oxidation of 1 µmol of D2HG per min.

### 2.4. Construction of MXene-Based Biosensor

Here, we developed a simple and convenient method to construct enzymatic electrodes. AuSPE was selected as the base electrode. Its length and width were 3 cm and 1 cm, respectively. The working electrode (4 mm diameter) and counter electrode were made of gold, and silver was used as the reference electrode. Before modification, the AuSPE was cleaned by ultrasonic in Milli-Q water and ethanol successively then was dried under a nitrogen stream. The biosensor was prepared as follows: firstly, 7.5 μL MB (100 mM, dissolved in water) and 40 μL enzyme solution (3 mg mL^−1^, dissolved in 20 mM PB) were added to 40 μL MXene solution (0.25 mg mL^−1^, dissolved in water). The mixture was shaken at 4 °C for 20 min to facilitate the adsorption of MB and *Rs*D2HGDH onto the surface of MXene. Then, 30 μL 0.5% chitosan solution was injected into the above mixture. Finally, 10 μL of the above-prepared mixture was applied to the AuSPE. As the electrode was slowly dried at room temperature, a uniform enzyme film was formed on its surface. Other enzymatic electrodes as controls were prepared by a similar method. All electrodes were immersed in 50 mM PB (pH 7.0) for 30min before use to remove unbound MB and *Rs*D2HGDH.

### 2.5. Construction of MXene-Based Biosensor

The CV curves were collected with a potential window from −0.4 V to 0 V at a scan rate of 10 mV s^−1^. I–t was measured at a fixed potential value of −0.23 V. The electrochemical impedance spectroscopy (EIS) was carried out in 5 mM K_3_/K_4_Fe(CN)_6_ solution with 0.1 M KCl at the open circuit potential. The AC frequency range was 100 kHz to 0.1 Hz, and the sinusoidal perturbation was 5 mV. Unless otherwise specified, the 50 mM PB (pH 7.0) with 0.1 M KCl was used as the supporting electrolyte for electrochemical experiments, which shall be purged with nitrogen for 30 min to remove oxygen and avoid its interference for electrochemical analysis.

## 3. Results

### 3.1. Characterization of MXene

**Figure 1:**
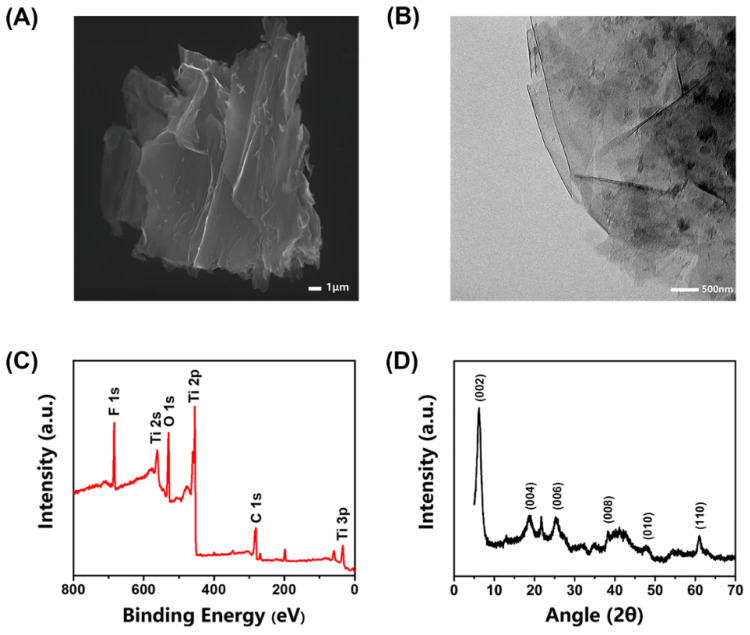
Characterizations of the synthesized MXene. (**A**) SEM image of MXene; (**B**) TEM pattern of MXene; (**C**) XPS survey scan of MXene; (**D**) XRD of the synthesized MXene.

The structure of MXene was characterized using SEM and TEM. Figure 1A,B shows that the synthesized MXene exhibits a graphene-like lamellar multilayer nanostructure after ultrasonic exfoliation, which provides a large number of enzyme attachment sites and the *Rs*D2HGDH molecules could be absorbed by surface functional groups of MXene with non-covalent interactions [37]. The XPS spectrum confirms the existence of elements C, O, F, and Ti (Figure 1C) in the synthesized MXene [44]. The core-level spectrums of various elements of XPS were fitted through relevant software (XPSpeak41) to further analyze the chemical bond of the element. The spectrum of the C 1s region was mainly fitted to three main peaks (Figure S1A), corresponding to C–Ti, C–C, and C–O bonds, respectively. In addition, there was a weak peak corresponding to the groups of O–C=O/C–F at 288.9 ev. The O1s region spectrum was fitted by five peaks, which were assigned to O–Ti/OH, O–C/OH, O–Ti, C=O, and adsorbed H_2_O, respectively, according to the corresponding binding energy (Figure S1B). The spectrum of the Ti 2p region was relatively complex, which was fitted by seven peaks. Among them, three pairs of peaks were assigned to Ti^2+^, Ti^3+,^ and Ti–C respectively, and a single peak corresponded to Ti–O/Ti–F (Figure S1C). These peaks indicate abundant functional groups on the surface of synthesized MXene. The peaks fitting is in good agreement with the results reported previously [45,46]. Next, the XRD of MXene was used to analyze the crystal structure at a scanning range of 5−80°. Figure 1D clearly shows that the MXene has an obvious characteristic peak at 6.2° [47]. Usually during the etching procedure, there were many unpaired electrons on the surface of MXene, tending to combine with other groups, such as –O, –F, and –OH [48]. The MXene was prepared in an aqueous phase, so its surface was rich in hydroxyl groups, which results in much better dispersion of MXene in the aqueous phase than that of carbon materials. This feature brings great convenience and many benefits for its application in our biosensor.

### 3.2. Characterization of RsD2HGDH

Different from the NAD^+^-dependent D2HGDH that is widely applied in the commercial assay kit, *Rs*D2HGDH can shuttle electrons to the electrode directly through many electron mediators with the assistance of its inherent flavin adenine dinucleotide (Figure S2A). In order to study the enzymatic properties and kinetic parameters in detail, we investigated the specific enzyme activity, *K_m_*, *K_cat_*, optimum temperature, and pH of *Rs*D2HGDH. Firstly, the specific enzyme activity of *Rs*D2HGDH was determined as 4.92 U mg^–1^ at 25 °C in 50 mM PB (pH 7.0). The *K_m_* of *Rs*D2HGDH for D2HG was 0.433 mM by measuring the corresponding enzymatic reaction rates under different concentrations of D2HG and the *K_cat_* for D2HG was estimated as 4.86 s^−1^ according to the maximum reaction rate (Figure S2B). In addition, we measured the relative activities of *Rs*D2HGDH for different substrates, including D/L2HG, D/L-lactate, D/L-malate, D/L-tartrate. Surprisingly, *Rs*D2HGDH showed the highest catalytic efficiency toward D2HG and low activity for L2HG, while almost no activity for other 2-hydroxycarboxylic acids, illustrating its excellent substrate specificity (Figure S2C). Then, the relative activity at different temperatures and pHs were measured in order to determine the optimal reaction conditions of *Rs*D2HGDH (Figure S2D,E). The relative activity reached the maximum value at 45 °C and began to decrease because of the inactivation of *Rs*D2HGDH and the optimum value of pH was found at pH 7.0. We also measured the thermal stability of *Rs*D2HGDH at 25 °C in the free state and the immobilized state respectively. As shown in Figure S3, the half-life of free *Rs*D2HGDH at 25 °C was 40.1 h. When it was immobilized on the electrode, its stability was improved, still maintaining 52% catalytic activity after 60 h. This could be attributed to the abundant functional groups on the surface of MXene that provide a favorable immobilization environment for the *Rs*D2HGDH, help it stabilize the tertiary structure, and improve its thermal stability [49,50]. For operation convenience, room temperature (25 °C) was chosen as the working temperature of the biosensor. Meanwhile, according to the optimum pH of the *Rs*D2HGDH, 50 mM PB (pH 7.0) with 0.1 M KCl was selected as the supporting electrolyte of the biosensor.

### 3.3. Construction of D2HG Biosensor

**Figure 2:**
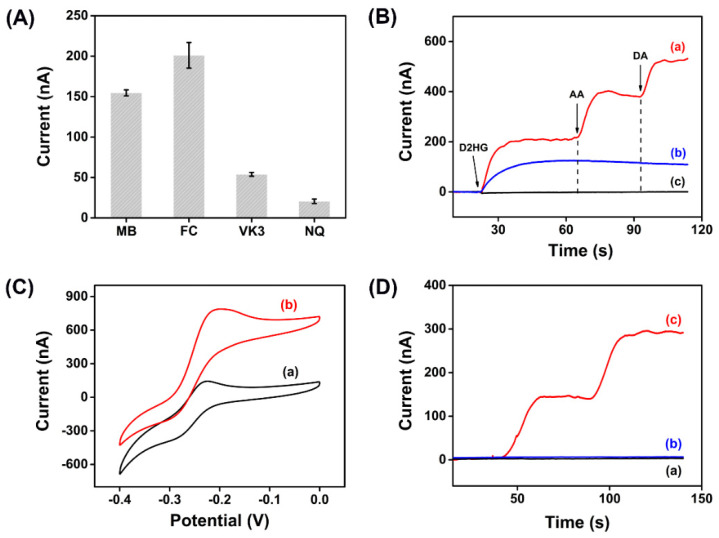
(**A**) Current response of enzymatic electrodes with different electron mediators for 50 μM D2HG in 50 mM PB (pH 7.0) with 0.1 M KCl; (**B**) i–t curves of enzymatic electrodes with (a) FC, (b) MB and (c) no mediator in 50 mM PB with 0.1 M KCl after adding 50 μM D2HG, 200 μM ascorbic acid (AA) and 200 μM dopamine (DA), respectively; (**C**) CV curves of the *Rs*D2HGDH/MB/MXene/AuSPE with (a) 0 μM and (b) 1 mM D2HG in 50 mM PB (pH 7.0) with 0.1 M KCl at a scan rate of 10 mV s^–1^ vs. Ag/AgCl; (**D**) i–t curves of the (a) AuSPE, (b) MXene/MB/AuSPE and (c) *Rs*D2HGDH/MB/MXene/AuSPE in 50 mM PB (pH 7.0) with 0.1 M KCl with the stepwise addition of 50 μM D2HG. Applied potential: −0.23 V vs. Ag/AgCl.

Electrochemical enzymatic biosensors are based on the electron transfer between enzyme and electrode, converting the microscopic biochemical reaction into the current signal. However, same as most enzymes in nature, *Rs*D2HGDH is unable to transfer electrons to the electrode directly due to the redox center deeply buried in the insulating protein shell. Accordingly, in this study, electron mediators were applied to promote electron transfer. Firstly, we compared the performance of different electron mediators on the current response of the enzymatic electrodes. Methylene blue (MB), ferrocene (FC), vitamin K_3_ (VK_3_), and 1,4-naphthoquinone (NQ) were used to prepare enzymatic electrodes and their CV curves were shown in Figure S4. Compared with the absence of D2HG, in the presence of 1 mM D2HG, the oxidation peak current increased significantly. Then, the current responses of enzymatic electrodes with different electron mediators were analyzed in detail by the i–t curve. As displayed in Figure 2A, FC had the maximum current response, followed by MB, and NQ had the worst effect. As well known, different electron mediators could affect the optimal working potential of biosensors. In this study, the oxidation peak potential of FC was more positive than MB, which may lead to the oxidation of many other electrochemically active substances and cause interference to the biosensor signal. As expected, when 200 μM ascorbic acid (AA) and 200 μM dopamine (DA) were added, obvious oxidation currents were observed with an FC enzymatic electrode while it generated no current response with an MB enzymatic electrode used. At the same time, the enzymatic electrode without any electron mediator had no current response to D2HG (Figure 2B). Therefore, we chose MB as the electron mediator for the following experiments. Next, CVs and i–t curves were used to perform the proof-of-concept for the D2HG assay. With the addition of 1 mM D2HG to the surface of the biosensor, a significant increase of oxidation current was observed in the CV curve (Figure 2C). Then, the bare AuSPE (AuSPE), the AuSPE modified by MXene and MB (MXene/MB/AuSPE) and the AuSPE modified by MXene, MB, and *Rs*D2HGDH (*Rs*D2HGDH/MXene/MB/AuSPE) were measured at −0.23 V (vs. Ag/AgCl) potential with the stepwise addition of 50 μM D2HG (Figure 2D). As expected, the *Rs*D2HGDH/MXene/MB/AuSPE generated an obvious oxidation current compared to the bare AuSPE and MXene/MB/AuSPE, which indicated that the oxidation current in the experiment came from the oxidation of D2HG catalyzed by *Rs*D2HGDH. 

In order to verify the stability of MB on the electrode surface, we soaked the prepared electrodes in 50 mM PB (pH 7.0) and investigated the immobilization efficiency of MB on the electrode surface by detecting the concentration of MB in the buffer at different times. MB had an obvious absorption peak at 664 nm and linearly correlated to its concentration (Figure S5A). Therefore, the concentration of MB can be calculated indirectly by the absorbance value at 664 nm. As shown in Figure S5B, a portion of the unbound MB was eluted within 10 min. Then, the amount of MB on the electrode remained stable, and the immobilization efficiency was about 44%. In addition, we conducted continuous CV scanning of the *Rs*D2HGDH/MB/MXene/AuSPE in the potential window of –0.4 V to –0.1 V to investigate the stability of MB in the electrochemical test (Figure S5C,D). In the first ten scans, the peak potential and peak current of MB were relatively stable. With the increase of the number of scanning cycles, the peak potential shifted positively, and the peak current increased slightly, which may be caused by the formation of polymethylene blue after electropolymerization of MB [51,52].

**Figure 3:**
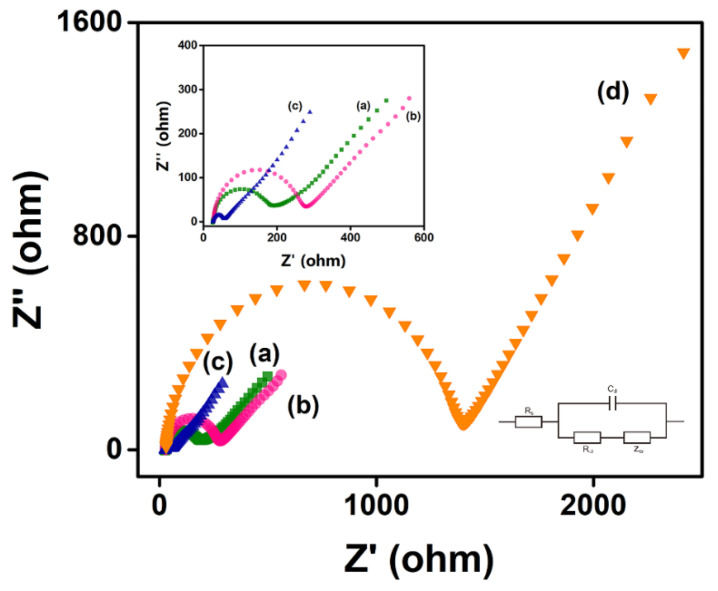
Nyquist plots of (a) AuSPE, (b) Chi/AuSPE, (c) Chi/MXene/AuSPE, (d) Chi/MXene/*Rs*D2HGDH/AuSPE in 5 mM K_3_/K_4_Fe(CN)_6_ redox probe containing 0.1 M KCl. Insert: Amplified Nyquist plot of (a) AuSPE, (b) Chi/AuSPE, (c) Chi/MXene/AuSPE.

The Nyquist plot of the electrode, which is modeled by Randles’s equivalent circuit, mainly has two parts: the low-frequency region representing the diffusion process and the high-frequency region representing the electron transfer resistance (*R*_et_), which shows an image similar to a semicircle, and its diameter is equal to the *R*_et_ of the electrode [53]. The bare AuSPE had a small *R*_et_, which increased slightly when chitosan was modified on the surface. When chitosan and MXene were modified on its surface at the same time, the *R*_et_ was significantly reduced, due to the excellent metal conductivity of MXene. When *Rs*D2HGDH was further deposited on the electrode, the *R*_et_ increased sharply (Figure 3). This can be attributed to the insulating enzyme membrane, which prevents the contact between the redox probe Fe(CN)_6_^3–/4–^ and electrode [54]. Apparently, the results of EIS show that MXene and *Rs*D2HGDH were successfully modified on the electrode.

### 3.4. Optimization of Experiment Parameters

In order to obtain higher sensitivity and a wider linear range of the biosensor, the amounts of MXene, MB, and *Rs*D2HGDH were further optimized. The i–t curves were applied to study electrochemical performance and the current responses of the biosensor with 50 μM D2HG under different preparation conditions were taken for comparison. Firstly, various amounts of MXene were applied to the biosensor (Figure S6A). With MXene amount increasing from 0.5 μg to 3 μg, the current response yields the maximum value at 1 μg. However, with the amount further increasing, the current response of the biosensor decreases gradually, which is attributed to the blockage of the electron transport path with excessive materials [40]. Next, the biosensors with different amounts of MB from 10 nmol to 100 nmol were analyzed (Figure S6B). With the increase of the amount of MB, the current response gradually increases and stabilizes until the amount of MB reaches 75 nmol, suggesting that the overloading of MB would barely boost the current signal of the biosensor. Finally, the optimal amount of *Rs*D2HGDH was determined. As shown in Figure S6C, the maximum value appears when the amount of *Rs*D2HGDH reaches 60 mU, and further increasing enzyme loading would cause the insulation effect of the enzyme membrane and hinder electron transfer and mass transfer processes at the electrode interface. Therefore, 1 μg MXene, 75 nmol MB, and 60 mU *Rs*D2HGDH were chosen as the optimal preparation condition for the D2HG biosensor.

### 3.5. Electrochemical Measurement

**Figure 4:**
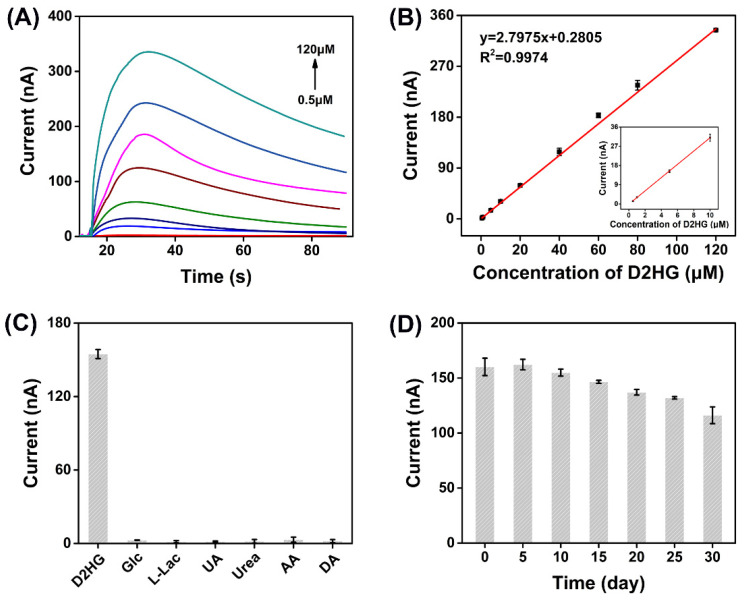
(**A**) I–t curves of the prepared biosensors in 50 mM PB (pH 7.0) with 0.1 M KCl containing 0.5, 1, 5, 10, 20, 40, 60, 80, 120 μΜ D2HG. Applied potential: −0.23 V vs. Ag/AgCl; (**B**) typical calibration plot for D2HG detection using the D2HG biosensors in 50 mM PB with 0.1 M KCl containing various concentrations of D2HG from 0.5 μΜ to 120 μΜ; (**C**) current response of biosensor in 50 mM PB (pH 7.0) with 0.1 M KCl containing 50 μΜ D2HG and some of the common interfering molecules, 5 mM Glc, 1 mM L-Lac, 300 μM UA, 30 mM urea, 200 μM AA and 200 μM DA. Applied potential: −0.23 V vs. Ag/AgCl; (**D**) stability of the D2HG biosensor in 50 mM PB (pH 7.0) with 0.1 M KCl. Applied potential: −0.23 V vs. Ag/AgCl.

The current responses of D2HG with different concentrations were recorded by i–t curves (Figure 4A). There was a typical linear correlation between the change of the oxidation current (Δ*i_p_*) and D2HG concentration in the range of 0.5 μM to 120 μM. The regression equation was calculated as Δ*i_p_* = 2.7975*c_D2HG_* + 0.2805 with a correlation coefficient of R^2^ = 0.9974. The sensitivity of the biosensor was 22.26 μA mM^–1^ cm^–2^ and the LOD was estimated as 0.1 μM (S/N = 3) by gradually reducing the concentration of D2HG (Figure 4B). The response time of the biosensor was about 15 seconds. Additionally, the specificity of the biosensor was researched by adding several common electroactive substances to the biosensor, including 5 mM glucose (Glc), 1 mM L-lactic acid (L-Lac), 300 μM uric acid (UA), 30 mM urea, 200 μM AA, and 200 μM DA, and almost no response current was generated, indicating the prepared D2HG biosensor has the strong anti-interference ability (Figure 4C). This merit could be attributed to the excellent substrate specificity of *Rs*D2HGDH and a negative working potential applied (−0.23 V vs. Ag/AgCl) which would not cause the oxidation of many substances. The repeatability was assessed by recording the current responses of five prepared D2HG biosensors. The relative standard deviation (RSD) of detection results was 2.65% (Figure S7), indicating fairly good repeatability. Furthermore, the D2HG biosensor still retained 72.52% performance of its incipient state after storage at 4 °C for 30 days, which demonstrates its gratifying storage stability (Figure 4D).

### 3.6. Real Sample Analysis

In order to further evaluate the performance of D2HG biosensor for real sample analysis, the recovery tests were studied by adding D2HG standard into fetal bovine serum and artificial urine samples. In patients with D2HGA, the concentration of D2HG in blood is around 60 μM, and it could be up to 1.5 mM in urine [55]. The fetal bovine serum and artificial urine obtained were added with D2HG standard with final concentrations of 20, 40 and 80 μM to the fetal bovine serum, and 1, 1.5 and 2 mM to the artificial urine, respectively. The artificial urine samples were diluted 10 times by 50 mM PB (pH 7.0) with 0.1 M KCl before the measurement.

**Figure 5:**
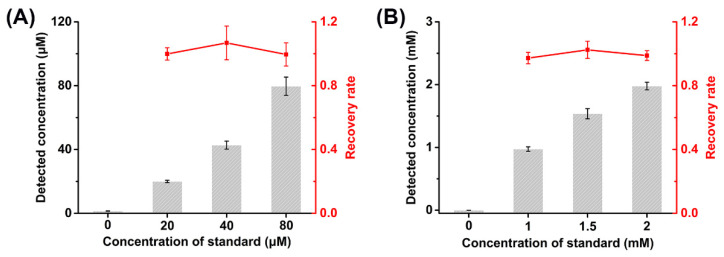
Recovery tests of (**A**) fetal bovine serum and (**B**) artificial urine.

The prepared biosensors were used to analyze the fetal bovine serum and artificial urine samples containing D2HG standard (Figure 5). It could be found that the recoveries of the fetal bovine serum samples were 99.56%–106.83% and those of artificial urine samples were 97.30%–102.47%. Meanwhile, the RSD numbers were less than 7.21% and 5.25%, respectively. The results indicate the D2HG biosensor based on *Rs*D2HGDH modified MXene had excellent accuracy and reliability, allowing it for the rapid and efficient detection of real samples.

## 4. Discussion

In this study, we cloned and expressed a new source of D2HGDH to avoid the use of expensive and unstable coenzymes (such as NAD^+^) in this biosensor. The successful preparation and application of MXene, due to its high hydrophilicity and rich surface functional groups, render favorable conditions for enzyme immobilization, making the D2HG biosensor have better performance than other reported methods (Table 1). For example, the detection limit of the commercial D2HG assay kit is about 10 μM [56], much higher than the concentration of D2HG in normal human serum (about 1 μM), so its measurement accuracy is limited. At the same time, the detection process of the kit needs to react continuously at 37 °C for 60 minutes, which seriously limits the detection speed. Alternatively, the fluorescence method based on resazurin analogs has a lower detection limit, but similarly, the detection process needs to control temperature and avoid light, and the detection speed is also slow. In addition, in order to ensure the accuracy of detection, some samples need complex pretreatment processes, such as protein removal, which makes the detection more cumbersome. In contrast, the D2HG biosensor developed in this work has no requirement for the detection environment, can carry out at room temperature, and features a fast detection speed (nearly 100 to 200 times faster than those abovementioned methods). In the meantime, there is almost no need to pretreat samples for the biosensor, and its excellent anti-interference ability further makes it work robustly in complex samples. Notably, the biosensor based on amplified luminescent proximity homogeneous assay was recently developed by Xu’s group which used DhdR, a specific transcriptional regulator, as a sensing element [57]. Compared with that sensor, our biosensor still has a considerable advantage in the simplicity of operation and detection speed. In addition, our work is not dependent on large-scale precision instruments, making it possible to detect D2HG intelligently and conveniently in vitro.

However, our biosensor still suffers from some limitations, for example, the competition between oxygen and mediators, the stability of mediators, and their biological toxicity. Therefore, future work should focus on the development of the third-generation biosensor by exploring new D2HGDH and bypassing the mediated electron transfer. In addition, we expect that the biosensor can be combined with portable intelligent equipment to realize the independent detection of molecules of interest.

## 5. Conclusions

In summary, we developed an electrochemical enzymatic biosensor for the diagnosis of D2HGA in vitro for the first time. Benefitting from the substrate specificity of *Rs*D2HGDH and the excellent conductivity of MXene, the biosensor had high sensitivity, good selectivity, low detection limit, favorable stability, repeatability, and wide linear range. The biosensor was also applied for the analysis of D2HG in fetal bovine serum and artificial urine samples and showed good accuracy and reliability. Compared with other commercial analyses of D2HG, our biosensor provides a simple, rapid, sensitive, and low-cost method for the detection and it is promising to be applied for the diagnosis of D2HGA and IDH-mutated cancers in the future.

## 6. Patents

Z.Z., B.W. and L.W. claim the financial interest to the technique disclosed in this study and have applied one Chinese patent (202111432786.0).

## Figures and Tables

**Table 1:** Comparison of the constructed D2HG biosensor with other methods.

Detection Method	Sensing Element	Coenzyme	LOD (μM)	Linear Range (μM)	Detection Time	Stability	Ref
Colorimetry	D2HGDH	NAD^+^ *	10	-	37 °C, 60 min	−20 °C, <60 days	assay kit
Fluorometry	D2HGDH	NAD^+^	2.2	1−100	37 °C, 60 min	-	[16]
Fluorometry	D2HGDH	NAD^+^	0.13	0.44–50	RT, 30 min	-	[17]
AlphaScreen **	DhdR ***	-	0.1	0.3–20	RT, 60 min	-	[36]
Amperometry	D2HGDH	FAD	0.1	0.5–120	RT, 15 s	4 °C, >30 days	this work

* NAD^+^: nicotinamide adenine dinucleotide. ** AlphaScreen: amplified luminescent proximity homogeneous assay. *** DhdR: a transcriptional regulator that can specifically recognize D2HG.

## Data Availability

Not applicable.

## Supplementary Materials

The following supporting information can be downloaded at: https://www.mdpi.com/article/10.3390/bios12020066/s1, Figure S1: Curve-fitted XPS spectrum related to (A) C 1s, (B) O 1s and (C) Ti 2p; Figure S2: (A) SDS-PAGE of purified *Rs*D2HGDH; (B) enzymatic reaction rates of *Rs*D2HGDH with different substrate concentrations; (C) selectivity of recombinant *Rs*D2HGDH for various substrates, including D/L2HG, D/L-lactate, D/L-malate and D/L-tartrate; (D) relative activity of *Rs*D2HGDH at different temperatures; (E) relative activity of *Rs*D2HGDH at different pHs; Figure S3: The thermal stability of free *Rs*D2HGDH and immobilized *Rs*D2HGDH at 25 °C. Figure S4: CV curves of enzymatic electrodes prepared with different electron mediators, (A) MB, (B) FC, (C) VK_3_ and (D) NQ, with (a) 0 μM and (b) 1 mM D2HG in 50 mM PB (pH 7.0) with 0.1 M KCl at a scan rate of 10 mV s^–1^ vs. Ag/AgCl; Figure S5: (A) Absorbance of full wavelength scanning with different concentrations of MB. Insert: calibration plot of absorbance value and MB concentration; (B) immobilization amount of MB on electrode surface at different elution times; (C) continuous cyclic voltammetry of the *Rs*D2HGDH/MB/MXene/AuSPE in 50 mM PB (pH 7.0) with 0.1 M KCl at a scan rate of 10 mV s^–1^ vs. Ag/AgCl; (D) the peak current and peak potential of continuous cyclic voltammetry. Figure S6: Optimization of the preparation conditions of biosensor, including the amount of (A) MXene, (B) MB and (C) *Rs*D2HGDH in 50 mM PB (pH 7.0) with 0.1 M KCl containing 50 μΜ D2HG. Applied potential: −0.23 V vs. Ag/AgCl; Figure S7: Repeatability of D2HG biosensor in 50 mM PB (pH 7.0) with 0.1M KCl containing 50 μΜ D2HG. Applied potential: −0.23 V vs. Ag/AgCl.
