# Supplementary material for: An Enzymatic Biosensor for the Detection of D-2-Hydroxyglutaric Acid in Serum and Urine
Source: Biosensors (Basel). 2022 Jan 25;12(2):66. doi: 10.3390/bios12020066 (PMC8869338; doi:10.3390/bios12020066)
Supplement: Supplementary file 1 [file biosensors-12-00066-s001.zip › biosensors-1545711-supplementary.pdf]

biosensors

Supplementary data

An Enzymatic Biosensor for the Detection of D-2-Hydroxyglutaric Acid in Serum and Urine

Bo Wu [1,2], Zehua Li [2,3], Zepeng Kang [2], Chunling Ma [2], Haiyan Song [2], Fuping Lu [1] and Zhiguang Zhu [2,3,4,*]

[1] Key Laboratory of Industrial Fermentation Microbiology, Ministry of Education, Tianjin Key Laboratory of Industrial Microbiology, The College of Biotechnology, Tianjin University of Science and Technology, No.9, 13th Avenue, Tianjin Economic and Technological Development Area, Tianjin 300457, China; wub@tib.cas.cn (B.W.); lfp@tust.edu.cn (F.L.)
[2] Tianjin Institute of Industrial Biotechnology, Chinese Academy of Sciences, 32 West 7th Avenue, Tianjin Airport Economic Area, Tianjin 300308, China; lizeh@tib.cas.cn (Z.L.); kangzp@tib.cas.cn (Z.K.); machl@tib.cas.cn (C.M.); songhy@tib.cas.cn (H.S.)
[3] University of Chinese Academy of Sciences, 19A Yuquan Road, Shijingshan District, Beijing 100049, China
[4] National Technology Innovation Center of Synthetic Biology, Tianjin 300308, China
* Correspondence: zhu_zg@tib.cas.cn

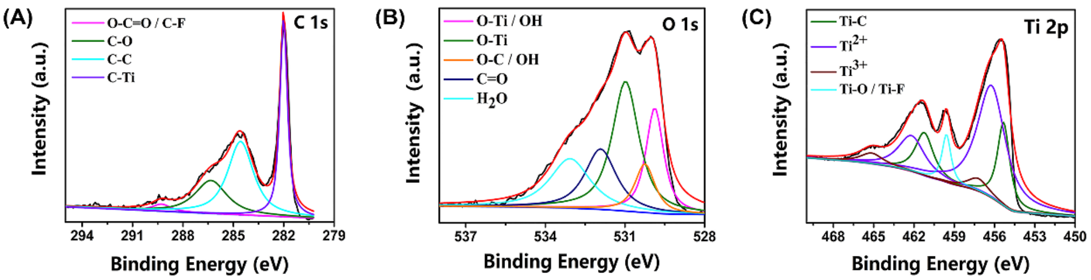

Figure S1. Curve-fitted XPS spectrum related to (A) C 1s, (B) O 1s and (C) Ti 2p.

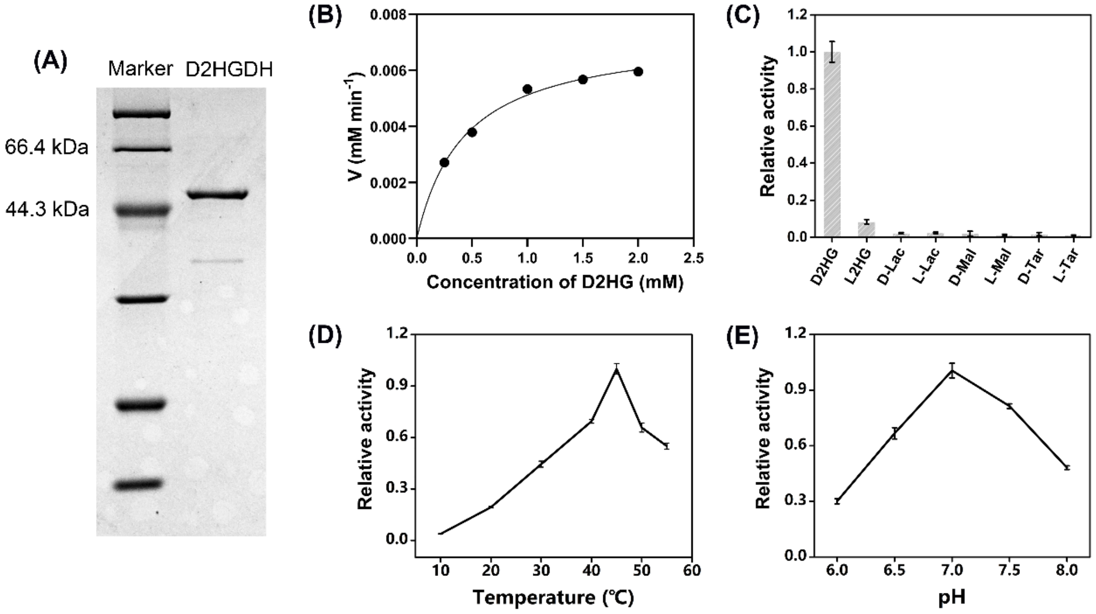

Figure S2. (A) SDS-PAGE of purified *Rs*D2HGDH; (B) enzymatic reaction rates of *Rs*D2HGDH with different substrate concentrations; (C) selectivity of recombinant *Rs*D2HGDH for various substrates, including D/L2HG, D/L-lactate, D/L-malate and D/L-tartrate; (D) relative activity of *Rs*D2HGDH at different temperatures; (E) relative activity of *Rs*D2HGDH at different pHs.

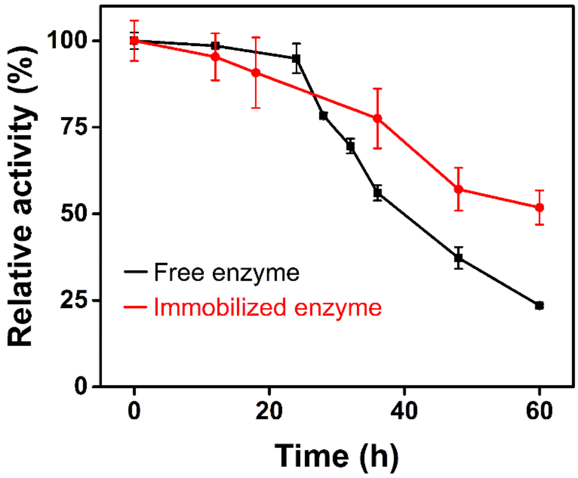

Figure S3. The thermal stability of free *Rs*D2HGDH and immobilized *Rs*D2HGDH at 25 °C.

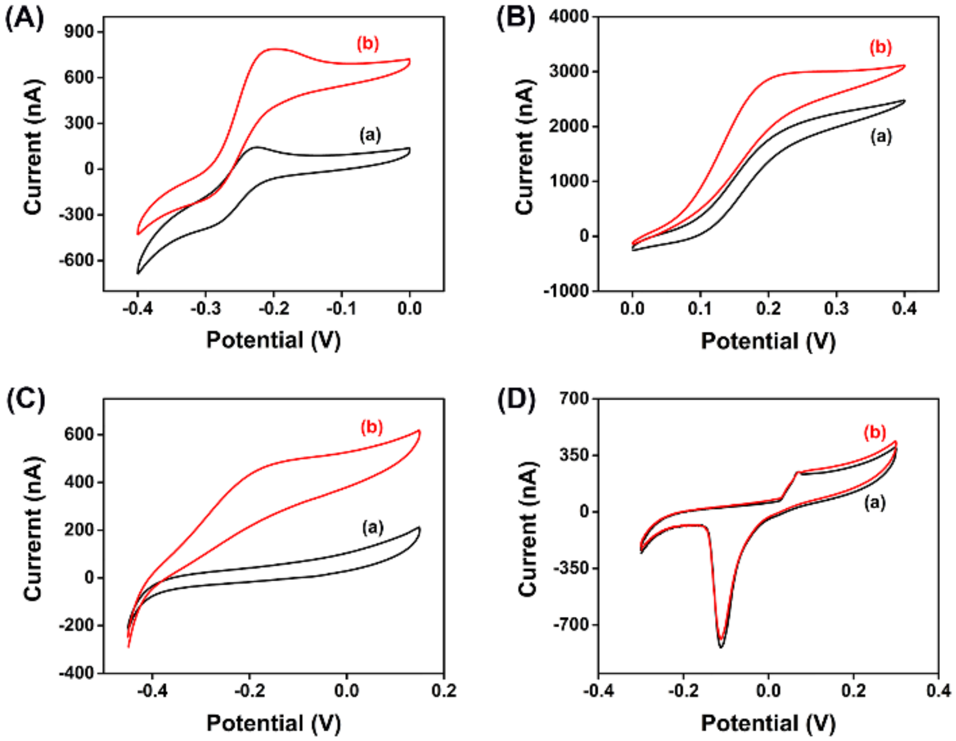

Figure S4. CV curves of enzymatic electrodes prepared with different electron mediators, (A) MB, (B) FC, (C) VK_3 and (D) NQ, with (a) 0 μM and (b) 1 mM D2HG in 50 mM PB (pH 7.0) with 0.1 M KCl at a scan rate of 10 mV s^{-1} vs. Ag/AgCl.

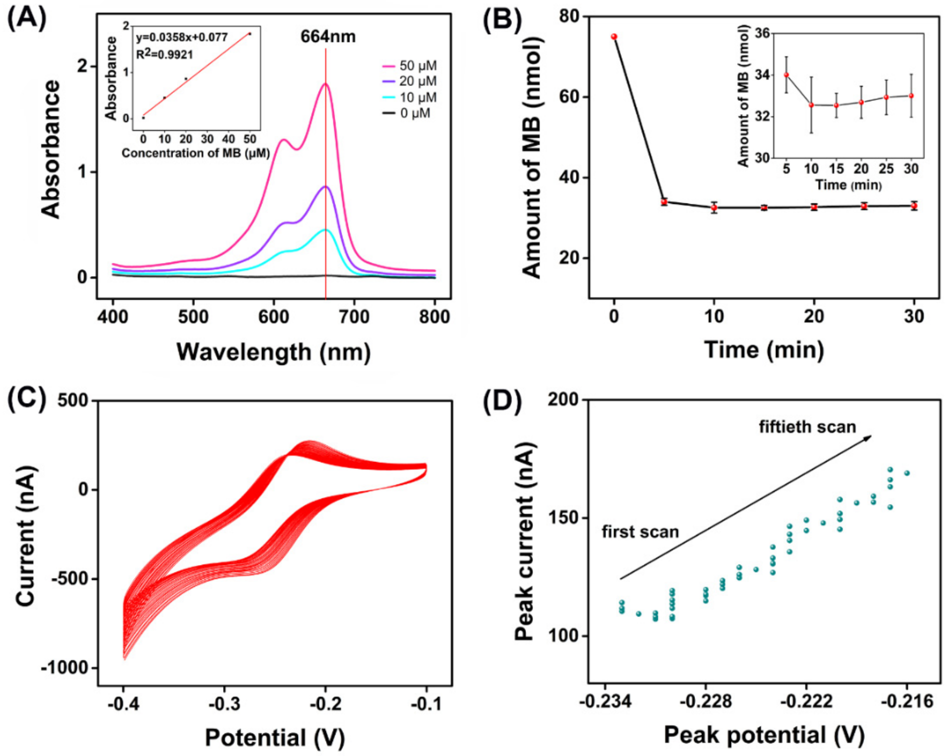

Figure S5. (A) Absorbance of full wavelength scanning with different concentrations of MB. Insert: calibration plot of absorbance value and MB concentration; (B) immobilization amount of MB on electrode surface at different elution times; (C) continuous cyclic voltammetry of the *Rs*D2HGDH/MB/MXene/AuSPE in 50 mM PB (pH 7.0) with 0.1 M KCl at a scan rate of 10 mV s^{-1} vs. Ag/AgCl; (D) the peak current and peak potential of continuous cyclic voltammetry.

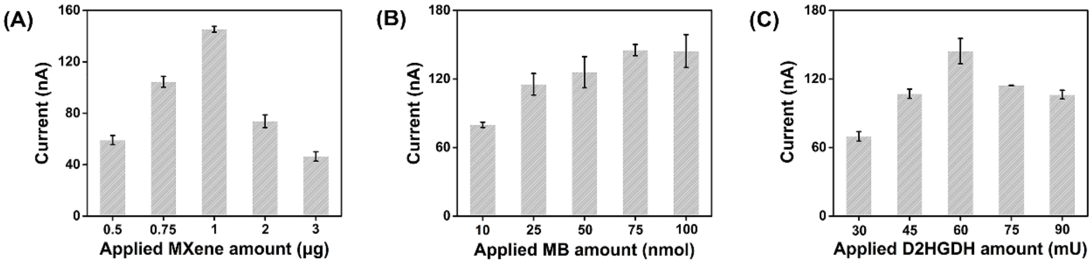

Figure S6. Optimization of the preparation conditions of biosensor, including the amount of (A) MXene, (B) MB and (C) *Rs*D2HGDH in 50 mM PB (pH 7.0) with 0.1 M KCl containing 50 μM D2HG. Applied potential: −0.23 V vs. Ag/AgCl.

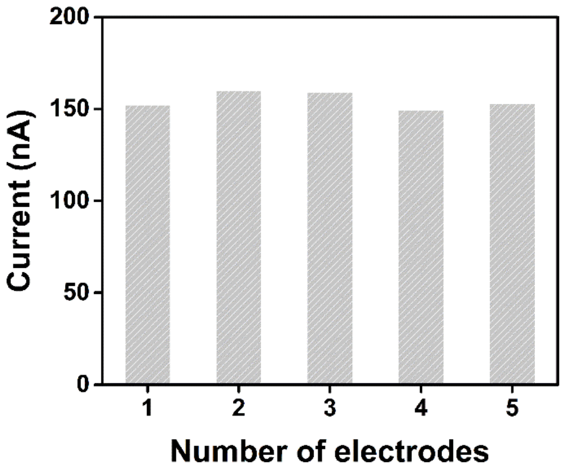

Figure S7. Repeatability of D2HG biosensor in 50 mM PB (pH 7.0) with 0.1M KCl containing 50 μM D2HG. Applied potential: −0.23 V vs. Ag/AgCl.
